# Risk factors for acute kidney injury after Stanford type A aortic dissection repair surgery: a systematic review and meta-analysis

**DOI:** 10.1080/0886022X.2022.2113795

**Published:** 2022-08-29

**Authors:** Lei Wang, Guodong Zhong, Xiaochai Lv, Yi Dong, Yanting Hou, Xiaofu Dai, Liangwan Chen

**Affiliations:** a Department of Cardiovascular Surgery, Union Hospital of Fujian Medical University, Fuzhou, China; b Key Laboratory of Cardio-Thoracic Surgery (Fujian Medical University), Fujian Province University, Fuzhou, China; c Department of Pathology, the Second People's Hospital, Fujian University of Traditional Chinese Medicine, Fuzhou, China; d Fujian Provincial Special Reserve Talents Laboratory, Fuzhou, China

**Keywords:** Type A aortic dissection, acute kidney injury, risk factors

## Abstract

**Background:** Risk factors for acute kidney injury (AKI) after Stanford type A aortic dissection (TAAD) repair are inconsistent in different studies. This meta-analysis systematically analyzed the risk factors so as to early identify the therapeutic targets for preventing AKI.

**Methods:** Studies exploring risk factors for AKI after TAAD repair were searched from four databases from inception to June 2022. The synthesized incidence and risk factors of AKI and its impact on mortality were calculated.

**Results:** Twenty studies comprising 8223 patients were included. The synthesized incidence of postoperative AKI was 50.7%. Risk factors for AKI included cardiopulmonary bypass (CPB) time >180 min [odds ratio (OR), 4.89, 95% confidence interval (CI), 2.06–11.61, *I^2^* = 0%], prolonged operative time (>7 h) (OR, 2.73, 95% CI, 1.95–3.82, *I^2^* = 0), advanced age (per 10 years) (OR, 1.34, 95% CI, 1.21–1.49, *I^2^* = 0], increased packed red blood cells (pRBCs) transfusion perioperatively (OR, 1.09, 95% CI, 1.07–1.11, *I^2^* = 42%), elevated body mass index (per 5 kg/m^2^) (OR, 1.23, 95% CI, 1.18–1.28, *I^2^* = 42%) and preoperative kidney injury (OR, 3.61, 95% CI, 2.48–5.28, *I^2^* = 45%). All results were meta-analyzed using fixed-effects model finally (*p* < 0.01). The in-hospital or 30-day mortality was higher in patients with postoperative AKI than in that without AKI [risk ratio (RR), 3.12, 95% CI, 2.54–3.85, *p* < 0.01].

**Conclusions:** AKI after TAAD repair increased the in-hospital or 30-day mortality. Reducing CPB time and pRBCs transfusion, especially in elderly or heavier weight patients, or patients with preoperative kidney injury were important to prevent AKI after TAAD repair surgery.

## Introduction

Acute Type A aortic dissection (TAAD) is the most critical condition in cardiovascular diseases with poor outcomes [1]. Despite the continuous improvement of surgical techniques and medical management, the morbidity and mortality after TAAD repair surgery still remain high [2]. Acute kidney injury (AKI) is one of the most common complications after TAAD repair surgery, with an incidence of 18–67% [3,4], which is higher than other cardiac surgeries [5]. What’s more, postoperative AKI in TAAD is related to increased in-hospital, short-term, and long-term mortality, as well as major complications [5–7]. However, no reliable drugs or specific treatments are available to prevent or cure AKI, except renal replacement therapy (RRT) [8]. But in fact, the prognosis of RRT after TAAD repair surgery is not ideal [9]. Therefore, early identification of risk factors for postoperative AKI is of great importance for reducing the incidence of AKI and improving the clinical outcomes of patients undergoing TAAD repair surgery. At present, some studies have analyzed the risk factors for AKI after TAAD repair surgery [5,6,10–27], but only one study was multicentric [23]. Besides, the sample size in most studies was not large enough and the results were inconsistent among different studies. In addition, there is no meta-analysis exploring risk factors for AKI after TAAD repair surgery, except one that investigated the independent risk factors for postoperative AKI and the impact of AKI on 30-day postoperative outcomes in patients with TAAD [7]. However, the retrieval strategy of this meta-analysis focused more on the impact of AKI on postoperative complications and mortality. Besides, the retrieval time range of this meta-analysis was too narrow which was from 2011 to 2017. Therefore, we conducted a systematic review and meta-analysis to perform a more completed retrieval and a more focused analysis of risk factors for AKI after TAAD repair surgery so as to make improved research in this field. The primary purpose of this meta-analysis was to comprehensively investigate the risk factors for AKI after TAAD repair surgery with multi-center data. The second purpose was to analyze the synthesized incidence of AKI and its impact on mortality after TAAD repair surgery. In this way, we try to provide better guidance for early identification and management of postoperative AKI in TAAD.

## Materials and methods

### Literature search strategy

This meta-analysis was reported according to the guidelines of the Meta-analysis Of Observational Studies in Epidemiology group [28] and Preferred Reporting Items for Systematic Reviews and Meta-Analyses (PRISMA) Protocols [29]. Electronic searches were performed using PubMed, Embase, Cochrane library and Web of science from their inception to June 2022 to analyze the synthesized incidence and risk factors of AKI and its impact on mortality after TAAD repair surgery. The search terms "Aneurysm, Dissection" and "Acute Kidney Injury" AND "Risk Factors" were used as key terms or Medical Subject Heading headings (Table S1 in Supplementary Material). The reference lists of retrieved articles were reviewed for further identification of potentially relevant studies. All identified articles were systematically assessed using the inclusion and exclusion criteria.

### Inclusion criteria

The approach of populations, interventions, comparisons, outcomes, and study designs (PICOS) was used to establish the inclusion criteria for our meta-analysis. Studies meeting the following criteria were included: (1) Study populations: Patients older than 18 years old who underwent repair surgery for TAAD. (2) Study intervention and comparators: Patients with or without AKI after TAAD repair surgery. (3) Study outcomes: The primary outcome was the risk factors for AKI after TAAD repair surgery. The secondary outcomes were the synthesized incidence of AKI and its impact on in-hospital or 30-day mortality. (4) Study type: Observational or randomized controlled trial studies that report odds ratio (OR) with 95% confidence interval (CI) or providing raw data which can calculate these values. (5) Definitions: The definitions of all risk factors were similar. The diagnostic criteria of AKI included three most commonly used criteria, that is RIFLE (Risk, Injury, Failure, Loss of function, End-stage renal disease), AKIN (Acute Kidney Injury Network) and KDIGO (Kidney Disease Improving Global Outcomes). AKI was diagnosed as the value of postoperative serum creatinine (sCr) 1.5-fold higher than the baseline level or an increased value of 0.3 mg/dl within 48 h postoperatively.

### Exclusion criteria

(1) Studies only analyze risk factors of postoperative acute renal failure, or continuous RRT (CRRT), or AKI of stage 2 or 3 after TAAD repair surgery. (2) Studies did not contain outcomes related to risk factors of postoperative AKI after TAAD repair surgery. (3) Review articles, conference abstracts, case reports, letters, editorials, animal studies, repetitive study or same population, articles not available for full texts, and non-English articles.

#### Data extraction and quality assessment

Two investigators (Lei Wang and Xiaochai Lv) independently reviewed the retrieved articles and evaluated the quality assessment. All data were extracted from article texts, tables, and figures and entered into Excel software (Microsoft, Bellingham, WA). Discrepancies between the 2 investigators were resolved by a senior investigator (Yanting Hou). When insufficient data were available from publications, corresponding authors were contacted to provide additional records if possible. Quality assessment was performed using the Newcastle Ottawa Scale, and a total score of ≥6 (of 9) was considered of high quality and low risk of bias [30].

#### Statistical analysis

R software (version 4.2.0, R Core Team, Vienna, Austria) was used for statistical analysis. The kappa statistic was calculated to assess inter-rater agreement at the title/abstract screening and full text review stages, with thresholds of ≥0.81 indicating substantial perfect agreement, as defined by McHugh [31]. Risk factors were synthesized analyzed if at least three studies reported such results. The pooled incidence rate, OR or risk ratio (RR) with 95% CI were calculated as summary statistics. Statistical heterogeneity was assessed between studies with Q statistic test and *I^2^* test [32]. *I^2^*<50% and *P* for heterogeneity >0.1 was considered low heterogeneity among studies and a fixed-effects model would be used for analysis. *I^2^*>50% and/or *P* for heterogeneity <0.1 denoted a significant heterogeneity among studies and a random-effects model would be used. The leave-one-out sensitivity analysis was performed to assess the influence of individual studies on the summary effect estimate, in which the meta-analysis estimates were computed by omitting one study at a time. Evidence of publication bias was explored through visual inspection of funnel plots (This applies to the inclusion of more than 10 articles), Egger’s test and Begg’s test [33,34]. It indicated no bias when funnel plot looked symmetrical, and Egger’s and Begg’s *p* > 0.05. The results with obvious bias with Egger's *p <* 0.05 and Begg's *p* < 0.05 were performed further by trim and fill adjustment. *p* < 0.05 was considered statistically significant. All *p* values were two-sided.

## Results

### Literature search results

**Figure 1.**
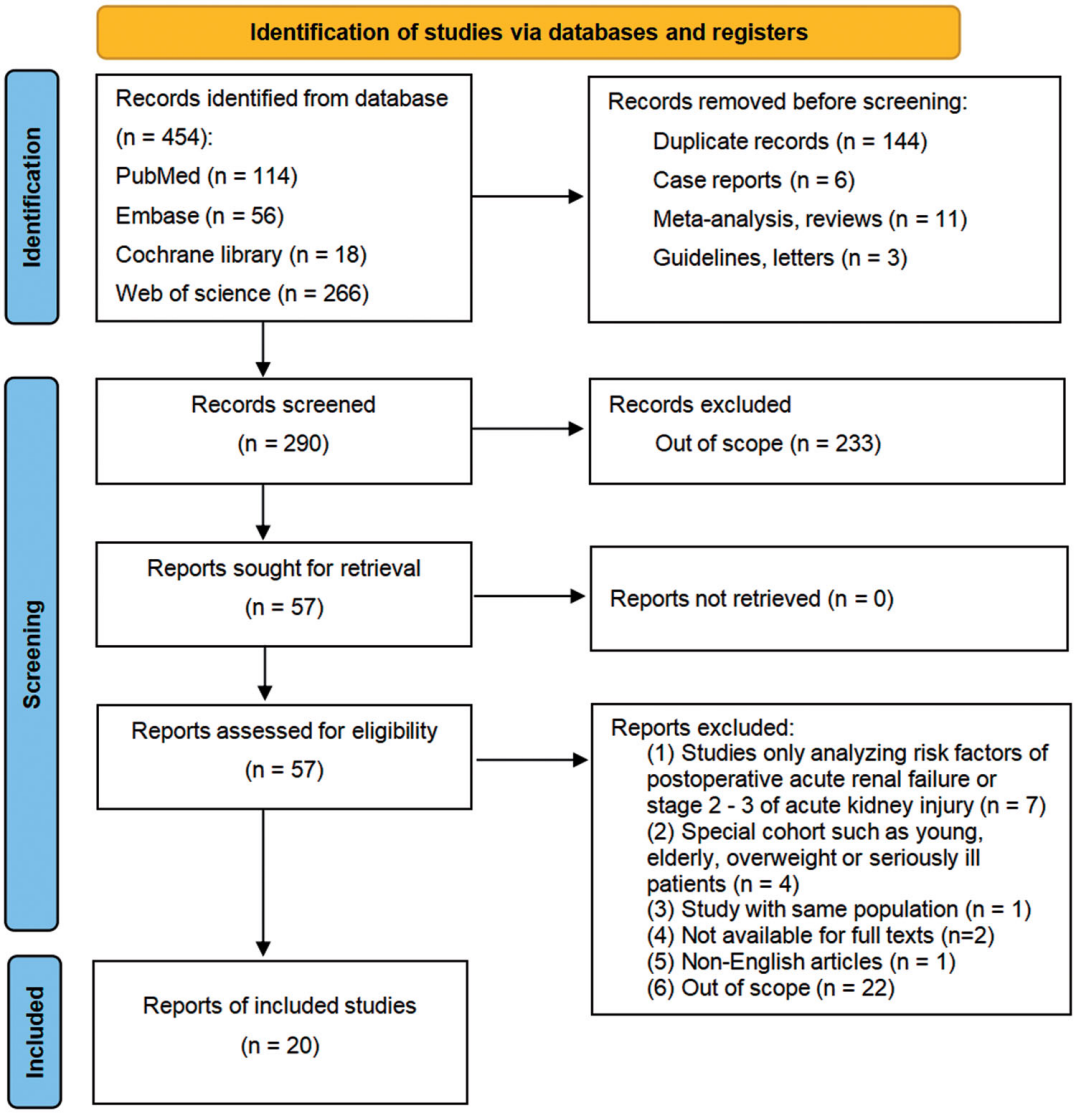
The PRISMA flow diagram of the present meta-analysis.

A total of 454 references were identified through four electronic database searches, and 20 studies were finally included for the present meta-analysis. The PRISMA flow diagram was shown in Figure 1. Inter-rater agreement was excellent at the screening (kappa= 0.828) and full-text review (kappa= 0.811) stages (Tables S2 and S3 in Supplementary Material). All studies reporting risk factors of postoperative AKI with OR and its 95% CI. Missing data of the incidence of CRRT and postoperative in-hospital or 30-day mortality were present in 2 and 5 included studies, respectively. These studies were excluded when such results were meta-analyzed for the missing data were few and they were all secondary outcomes.

### Study characteristics and quality assessment

Study characteristics are summarized in Table 1. Twenty included studies were all retrospective studies with two propensity score matching studies [13,19]. Thirteen studies emanated from China, three from Korea, two from Japan, and one each from Pennsylvania and Iceland. A total of 8223 patients undergoing aortic arch surgery were enrolled. Altogether 30 risk factors were involved, and six of them, that is prolonged cardiopulmonary bypass (CPB) time, prolonged operative time, advanced age, elevated body mass index (BMI), increased perioperative packed red blood cells (pRBCs) transfusion and preoperative kidney injury, were mentioned in three or more studies, which were further analyzed in this meta-analysis. All studies were assessed of high quality with ≥6 scores, except one study [25] with 5 scores that were considered low quality (Table S4 in Supplementary Material).

**Table 1.** Summary of study characteristics.

Study	Country	Study type	Diagnosis of patients	Case, n	mean age, years	Diagnostic criteria of AKI	AKI, n (%)	CRRT, n (%)	Risk factors, odds ratio (95% confidence interval)	30-day or in-hospital mortality	
										AKI group, n (%)	Non-AKI group, n (%)
Roh et al. (2012)	Korea	Retrospective, single center	thoracic AD	98	55 ± 15	RIFLE	53 (54%)	11 (11%)	CPB tim*e* > 180 min, 7.50 (1.70 − 33.17); preoperative elevated serum creatinine level, 8.43 (1.48 − 48.00).	1 (1%)	5 (11.1%)
Tsai et al. (2012)	China	Retrospective, single center	AD, 61.9% TAAD	268	53 ± 14	RIFLE	141 (52.7%)	30 (11.2%)	hypertension, 2.340 (1.104 − 4.959); sepsis 2.594 (1.031 − 6.526); postoperative lower limb malperfusion 4.558 (1.247 − 16.666).	31 (23.0%)	9 (7.4%)
Hiraoka et al. (2013)	Japan	Retrospective, single center	48.5% AD	200	71.8 ± 10.7	RIFLE	88 (44%)	9 (5%)	prolonged operative time (>490 min), 3.0 (1.5–6.2).	9 (10.2%)	2 (1.8%)
Kim et al. (2013)	Korea	Retrospective, single center	48.8% AD	417	57.9 ± 5.5	RIFLE	121 (29%)	24 (5.8%)	ag*e* ≥ 60 years, 1.83 (1.13 − 2.96); preoperative GF*R* < 60 mL/min/1.73 m^2^, 2.36 (1.40 − 3.96); LVE*F* < 55%, 2.08 (1.14 − 3.79); operation tim*e* > 7 h, 2.63 (1.63 − 4.24); intraoperative urine outpu*t* < 0.5 mL/kg/h, 2.81 (1.37 − 5.77); intraoperative furosemide use, 1.99 (1.25 − 3.16).	12 (2.7%)	7 (2.4%)
Kim et al. (2015)	Korea	Retrospective, PSM, single center	50.1% AD	702	58.4 ± 4.5	RIFLE	201 (28.6%)	42 (5.9%)	preoperative hemoglobi*n* < 10 g/dL, 2.58 (1.13–5.87)；preoperative albumi*n* < 4.0 g/dL, 2.50 (1.39–4.50)；intraoperative diagnosis of dissection, 2.44 (1.30–4.58)；operation tim*e* > 7 h, 2.71 (1.43–5.11)；DHC*A* > 30 min, 2.50 (1.18–5.28)；fresh frozen plasma use on surgery da*y* > 500 mL, 2.62 (1.33–5.20).	18 (9.0%)	19 (3.8%)
Qiu et al. (2015)	China	Retrospective, single center	TAAD	155	51.7 ± 8.7	AKIN	56 (36.1%)	10 (6.5%)	advanced age (per 10 years), 2.32 (1.47–3.68); stented elephant trunk implantation surgery, 3.29 (1.12–9.67); CPB tim*e* > 180min, 3.92 (1.35–11.35); pRBCs transfusio*n* > 10 U, 4.89 (2.03–11.76).	31 (1%)	6 (6%)
Ko et al. (2015)	Japan	Retrospective, single center	TAAD	375	66.4 ± 13.3	KDIGO	165 (44%)	33 (9%)	CPB time, 1.08 (1.02–1.14); high BMI (per 5 kg/m^2^), 1.58 (1.15–2.18); preoperative renal malperfusion, 9.06 (2.82–29.13); perioperative sepsis, 2.82 (1.19–6.66); perioperative peak C reactive protein, 1.06 (1.02–1.10).	6 (3.6%)	0
Ruan et al. (2016)	China	Retrospective, single center	TAAD	337	48.0 ± 11.0	KDIGO	152 (45.1%)	29 (8.6%)	postoperative lactate level at 6 h, 1.284 (1.129–1.460).	NR	NR
Arnaoutakis et al. (2016)	Pennsylvania	Retrospective, single center	aneurysmal disease	589	60.7 ± 13.1	RIFLE	86 (14.6%)	4 (0.6%)	longer CPB time, 1.01 (1.00–1.01).	2 (2.3%)	2 (0.41%)
Zhou et al. (2018)	China	Retrospective, single center	95% thoracic AD	553	46.5 ± 11.1	KDIGO	429 (77.6%)	63 (11.4%)	increased age (per 10 years), 1.37 (1.14–1.67); prolonged CPB time (per 30 min), 1.17 (1.01–1.37); elevated BMI (per 5 kg/m^2^), 1.41 (1.08–1.87); male sex, 1.94 (1.22–3.18).	11 (10.5%)	2 (1.6%)
Fang et al. (2019)	China	Retrospective, single center	TAAD	627	46.7 ± 10.5	KDIGO	473 (75.4%)	10 (1.6%)	advanced age, 1.02 (1.00–1.04); CPB duration, 1.01 (1.00–1.01); high BMI, 1.06 (1.01–1.12); hypertension, 1.76 (1.14–2.70).	11 (2.3%)	2 (1.3%)
Xu et al. (2019)	China	Retrospective, PSM, single center	TAAD	115	47.8 ± 10.7	KDIGO	61 (53%)	23 (20%)	CPB time, 1.171 (1.002–1.368).	NR	NR
Li L et al. (2020)	China	Retrospective, single center	TAAD	335	47.6 ± 10.3	KDIGO	241 (71.9%)	41 (12.2%)	CPB time, 1.008 (1.002–1.013); high BMI, 1.221 (1.148–1.245); perioperative pRBCs transfusion, 1.186 (1.055–1.334); preoperative chronic kidney disease, 12.352 (1.207–126.414); preoperative chronic liver disease, 2.207 (1.452–5.643); postoperative hypoproteinemia, 5.091 (1.855–13.976).	70 (21.2%)	32 (9.6%)
Liu Y et al. (2020)	China	Retrospective, single center	TAAD	130	54.7 ± 11.8	KDIGO	81 (63.1%)	NR	perioperative pRBCs transfusion (increase per 200 ml), 1.31 (1.01–1.71).	19 (23.5%)	2 (4.17%)
Wang Z et al. (2020)	China	Retrospective, single center	TAAD	712	52.5 ± 13.2	KDIGO	359 (50.4%)	111 (15.9%)	preoperative Cystatin C concentration, 2.92 (1.54–5.54).	65 (18.1%)	25 (7.1%)
Helgason et al. (2021)	Iceland	Retrospective, multicenter	TAAD	941	61.4 ± 12.0	RIFLE	382 (40.6%)	105 (11%)	advanced age (per 10 years), 1.3 (1.15–1.48); CPB time (per 10 min), 1.04 (1.02–1.07); BM*I* > 30kg/m2, 2.16 (1.51–3.09); preoperative renal malperfusion, 4.39 (2.23–9.07); preoperative other organ malperfusion, 2.1 (1.55–2.86); perioperative pRBCs transfusion,1.08 (1.06–1.1).	65 (17.0%)	37 (6.6%)
Tong et al. (2021)	China	Retrospective, single center	TAAD	660	48.6 ± 10.3	KDIGO	297 (45%)	NR	autologous platelet rich plasma, 1.729 (1.225–2.440).	NR	NR
Ma et al. (2021)	China	Retrospective, single center	TAAD	190	47 ± 14.8	KDIGO	131 (68.9%)	11 (5.8%)	CPB time, 1.06 (1.02–1.10); preoperative low Lymphocyte to Monocyte Ratio, 0.83 (0.71–0.98).	NR	NR
Li C et al. (2022)	China	Retrospective, single center	TAAD	421	47.7 ± 10.8	KDIGO	228 (54.2%)	65 (15.4%)	pRBCs transfusion, 1.11 (1.06–1.17); platelet concentrate transfusions, 1.28 (1.07–1.54).	NR	NR
Yang et al. (2022)	China	Retrospective, single center	TAAD	398	48.4 ± 9.8	KDIGO	268 (67.3%)	57 (14.3%)	increased serum myoglobin, 3.41 (1.67–6.98).	46 (17.2%)	1 (0.8%)

PSM: propensity score matched study; AD: aortic dissection; TAAD: Stanford type A aortic dissection; AKI: acute kidney injury, CRRT: continuous renal replacement therapy; RIFLE: Risk, Injury, Failure, Loss of function, End-stage renal disease; AKIN: Acute Kidney Injury Network; KDIGO: Kidney Disease Improving Global Outcomes; CPB: cardiopulmonary bypass; GFR: glomerular filtration rate; LVEF: left ventricular ejection fraction; DHCA: deep hypothermia circulatory arrest time; pRBCs: packed red blood cells; BMI: body mass index; NR: not reported.

### The incidence of AKI and CRRT after TAAD repair surgery

The synthesized incidence of AKI and CRRT after TAAD repair surgery was 50.7% (range, 43.3%–58.1%) and 9.1% (range, 6.9%–11.4%), respectively, which was similar to the previous studies (Figures S1 and S2 in Supplementary Material). Besides, subgroup analysis of risk factors of AKI was conducted based on different diagnostic criteria of AKI and different country research (Figures S3 and S4 in Supplementary Material). It showed that the incidence of AKI was higher in KIDGO diagnostic criteria than in AKIN or RIFLE criteria, suggesting that KIDGO criteria were more sensitive in diagnosing AKI. In addition, the incidence of AKI in Chinese studies was higher than in other countries, which might be related to a large deviation from the results of fewer study in other countries, and might be because most Chinese studies (11 out of 13) had used KIDGO diagnostic criteria which had higher sensitivity for AKI diagnosis, leading to a higher incidence of AKI.

### The risk factors for AKI after TAAD repair surgery

**Figure 2.**
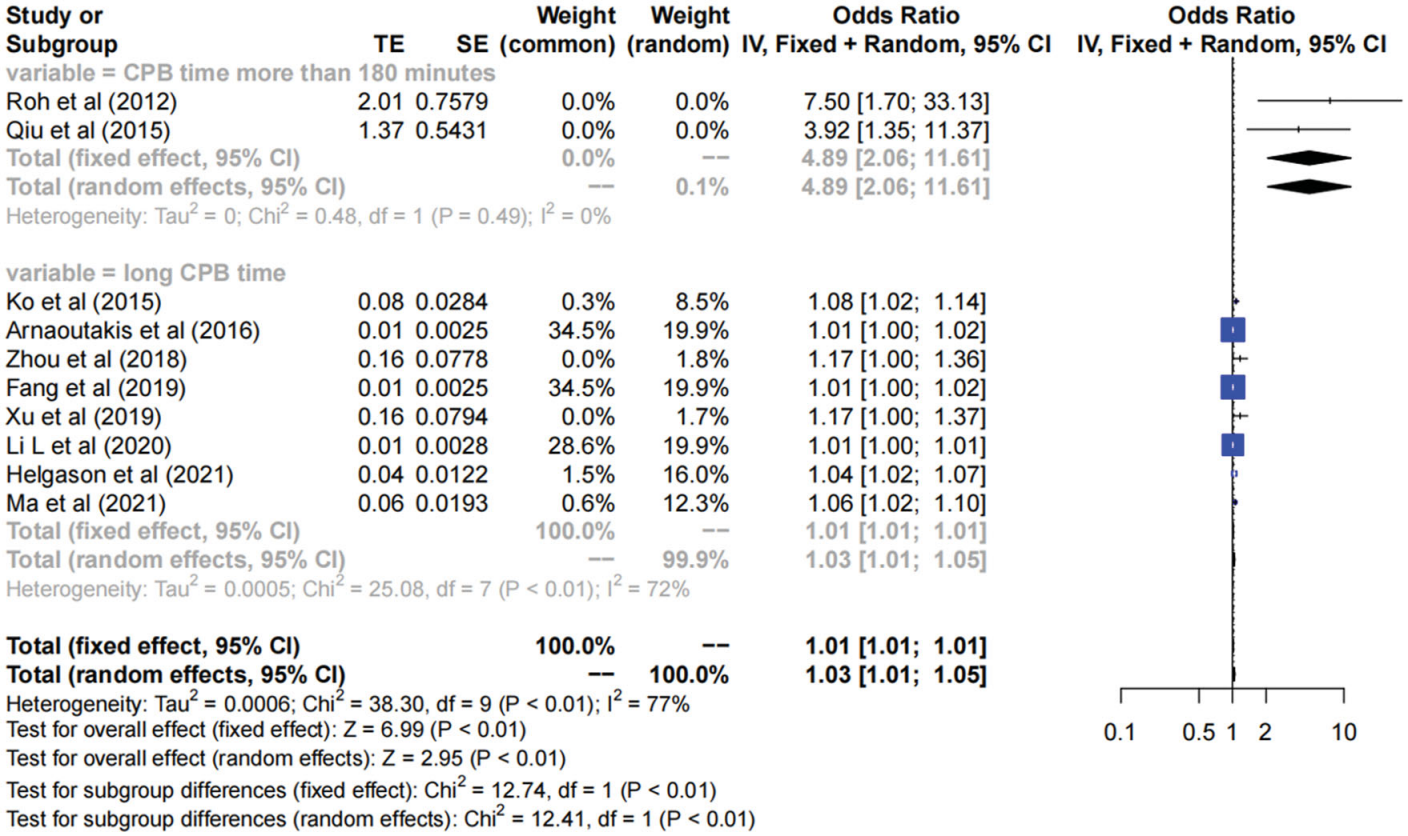
Forest plot of risk factor of prolonged CPB time. The solid squares are proportional to the weights used in the meta-analysis. The solid vertical line indicates no effect. The horizontal lines represent the 95% confidence interval (CI). The diamond indicates the weighted odds ratio, and the lateral tips of the diamond indicate the associated 95% CI. Fixed, fixed effects; Random, random effects.

**(1) Prolonged CPB time.** Ten studies [5,6,14,16–20,23,25] suggested that prolonged CPB time was a risk factor for AKI after TAAD repair surgery (OR, 1.03, 95%CI, 1.01–1.05, *I^2^*=77%, *P* for heterogeneity <0.01, *p* < 0.01) (Figure S5 in Supplementary Material). Since the definition of “prolonged CPB time” in the study of “Roh et al. (2012)” and “Qiu et al. (2015)” was “CPB time >180 min” which were different from other studies, subgroup analysis was further conducted according to the different definitions (Figure 2). The subgroup of “CPB time> 180 min” showed no heterogeneity (*I^2^*=0, *P* for heterogeneity >0.1), and the subgroup of “long CPB time” showed moderate heterogeneity (*I^2^*=72%, *P* for heterogeneity <0.01), so we concluded that prolonged CPB time, especially CPB time >180 min was a risk factor for AKI after TAAD repair surgery.

**Figure 3.**
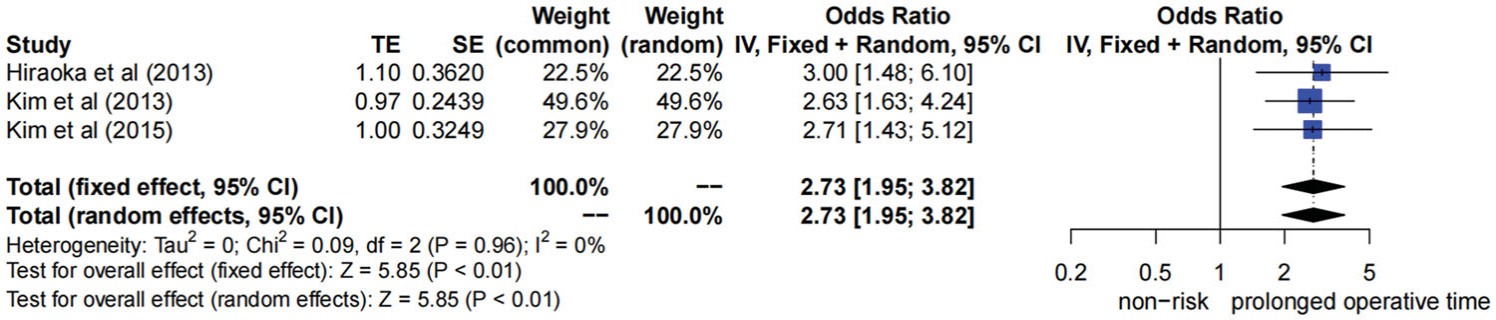
Forest plot of risk factor of prolonged operative time. The solid squares are proportional to the weights used in the meta-analysis. The solid vertical line indicates no effect. The horizontal lines represent the 95% confidence interval (CI). The diamond indicates the weighted odds ratio, and the lateral tips of the diamond indicate the associated 95% CI. Fixed, fixed effects; Random, random effects.

**(2) Prolonged operative time.** Three studies [11–13] suggested that prolonged operative time (>7 h) was a risk factor for AKI after TAAD repair surgery (*I^2^*=0, *P* for heterogeneity >0.1) (Figure 3), so fixed-effects model was used for analysis. The pooled OR (95% CI) was 2.73 (1.95–3.82) (*p* < 0.01), so we concluded that prolonged operative time (>7 h) was a risk factor for AKI after TAAD repair surgery.

**Figure 4.**
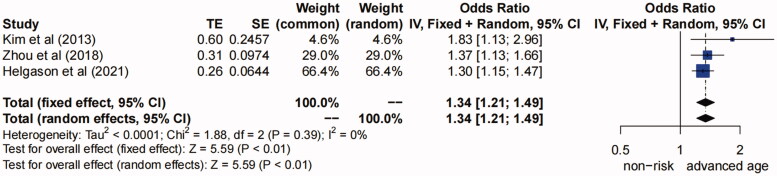
Forest plot of risk factor of advanced age. The solid squares are proportional to the weights used in the meta-analysis. The solid vertical line indicates no effect. The horizontal lines represent the 95% confidence interval (CI). The diamond indicates the weighted odds ratio, and the lateral tips of the diamond indicate the associated 95% CI. Fixed, fixed effects; Random, random effects.

**(3) Advanced age.** Five studies [12,14,17,18,23] suggested that the advanced age was a risk factor for AKI after TAAD repair surgery (*I^2^*=90%, *P* for heterogeneity <0.01, *p* < 0.01) (Figure S6 in Supplementary Material). Sensitivity analysis showed the study of “Qiu et al. (2015)” and “Fang et al. (2019)” might be the source of heterogeneity (Figure S7 in Supplementary Material), and after excluding these two studies, there was no heterogeneity between studies (*I^2^*=0%, *P* for heterogeneity >0.1) (Figure 4). Thus the fixed-effects model was used, and the pooled OR (95% CI) was 1.34 (1.21–1.49) (*p* < 0.01), so we concluded that the advanced age (per 10 years) was a risk factor for AKI after TAAD repair surgery.

**Figure 5.**
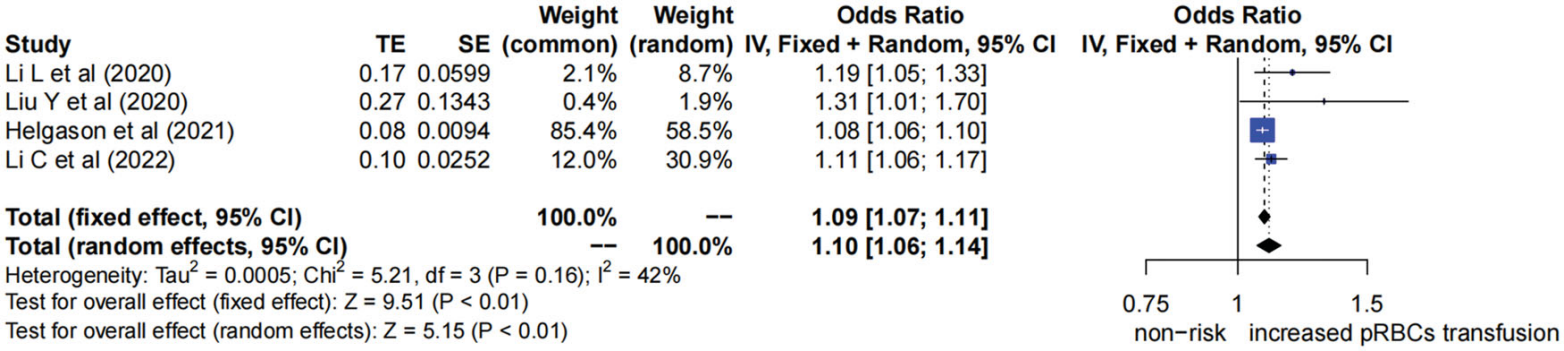
Forest plot of risk factor of increased pRBCs transfusion. The solid squares are proportional to the weights used in the meta-analysis. The solid vertical line indicates no effect. The horizontal lines represent the 95% confidence interval (CI). The diamond indicates the weighted odds ratio, and the lateral tips of the diamond indicate the associated 95% CI. pRBCs, packed red blood cells; Fixed, fixed effects; Random, random effects.

**(4) The increased pRBCs transfusion perioperatively.** Five studies [14,20,21,23,26] suggested that the increased pRBCs transfusion perioperatively was a risk factor for AKI after TAAD repair surgery. The definition in the study of “Qiu et al. (2015)” was “RBC transfusion >10 units” which was different from other studies, so this study was excluded. The forest plot of the remaining four studies showed low heterogeneity (*I^2^*=42%, *P* for heterogeneity >0.1), (Figure 5) and fixed-effects model was used. The pooled OR (95% CI) was 1.09 (1.07, 1.11) (*p* < 0.01), so we concluded that the increased pRBCs transfusion perioperatively was a risk factor for AKI after TAAD repair surgery.

**Figure 6.**
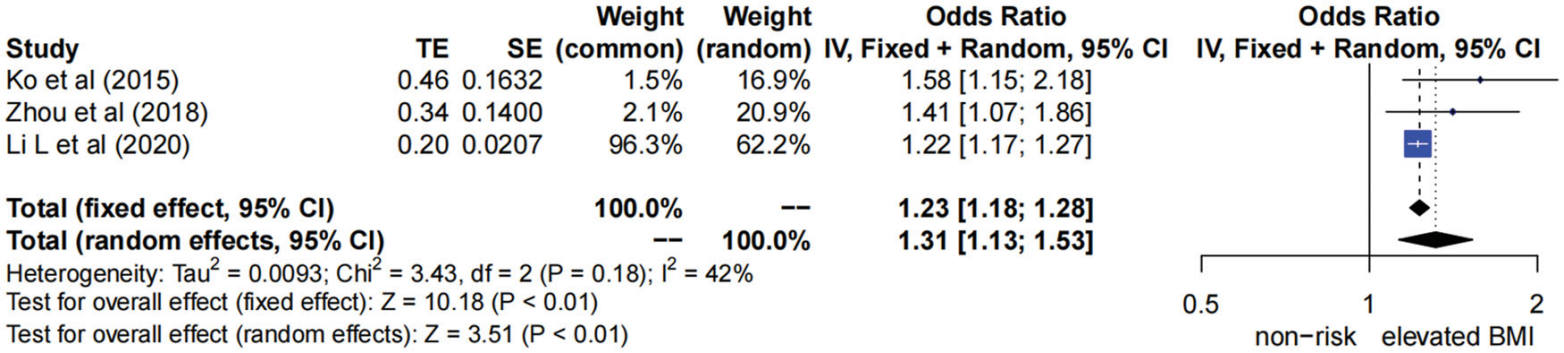
Forest plot of risk factor of elevated BMI. The solid squares are proportional to the weights used in the meta-analysis. The solid vertical line indicates no effect. The horizontal lines represent the 95% confidence interval (CI). The diamond indicates the weighted odds ratio, and the lateral tips of the diamond indicate the associated 95% CI. BMI, body mass index; Fixed, fixed effects; Random, random effects.

**(5) Elevated BMI.** Five studies [5,17,18,20,23] suggested that high BMI was a risk factor of AKI after TAAD repair surgery (*I^2^*=88%, *P* for heterogeneity <0.01, *p* < 0.01) (Figure S8 in Supplementary Material). Sensitivity analysis showed “Fang et al. (2019)” and “Helgason et al. (2020)” might be the source of heterogeneity (Figure S9 in Supplementary Material). After excluding these two studies, the heterogeneity between studies was lowered (*I^2^*=42%, *P* for heterogeneity >0.1) (Figure 6). Therefore, the fixed effects model was adopted for analysis, and the pooled OR (95% CI) was 1.23 (1.18, 1.28) (*p* < 0.01). So we concluded that elevated BMI (per 5 kg/m^2^) was a risk factor for AKI after TAAD repair surgery.

**Figure 7.**
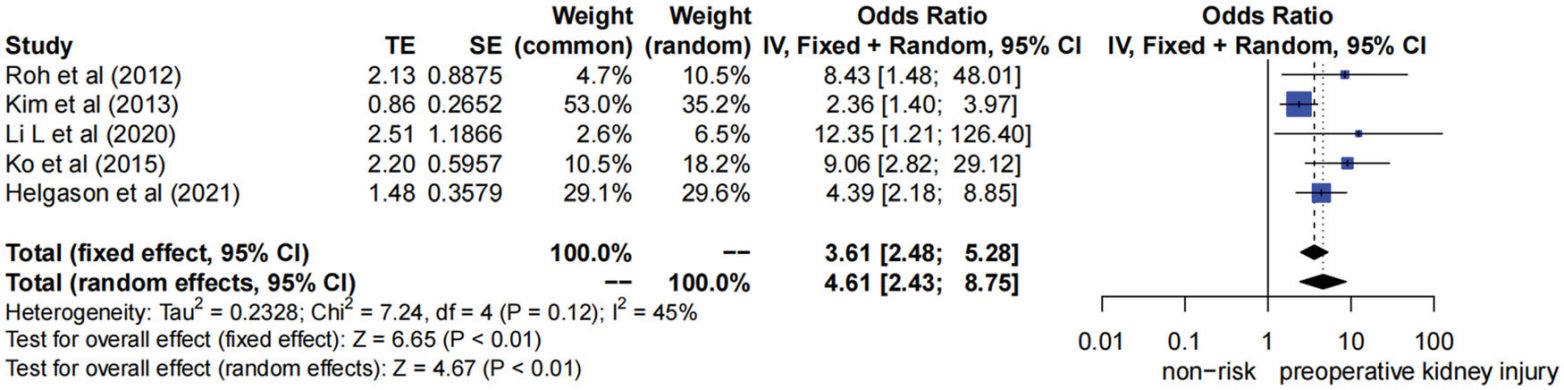
Forest plot of risk factor of preoperative kidney injury. The solid squares are proportional to the weights used in the meta-analysis. The solid vertical line indicates no effect. The horizontal lines represent the 95% confidence interval (CI). The diamond indicates the weighted odds ratio, and the lateral tips of the diamond indicate the associated 95% CI. Fixed, fixed effects; Random, random effects.

**(6) Preoperative kidney injury.** Five studies [5,6,12,20,23] suggested that preoperative elevated serum creatinine level, glomerular filtration rate <60 mL/min/1.73 m^2^, renal malperfusion, or chronic kidney disease were risk factors of AKI, and these risk factors are collectively classified as preoperative kidney injury. The forest plot showed low heterogeneity (*I^2^*=45%, *P* for heterogeneity >0.1) (Figure 7). Therefore, the fixed effects model was adopted, and the pooled OR (95% CI) was 3.61 (2.48–5.28) (*p* < 0.01), so we concluded that preoperative kidney injury was a risk factor for AKI after TAAD repair.

### The impact of AKI after TAAD repair surgery on mortality

**Figure 8.**
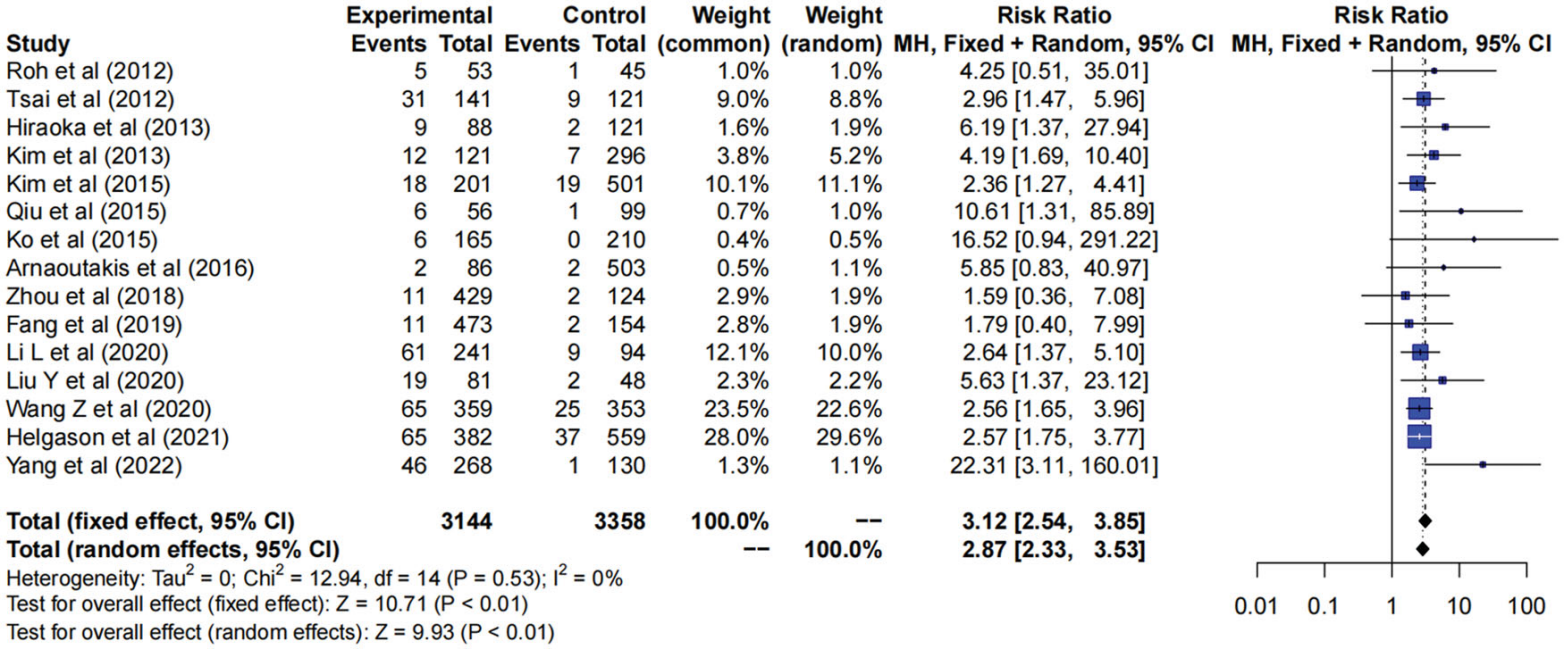
Forest plot of in-hospital or 30-day mortality in postoperative AKI and non-AKI group. The solid squares are proportional to the weights used in the meta-analysis. The solid vertical line indicates no effect. The horizontal lines represent the 95% confidence interval (CI). The diamond indicates the weighted odds ratio, and the lateral tips of the diamond indicate the associated 95% CI. Fixed, fixed effects; Random, random effects.

The in-hospital or 30-day mortality in patients with postoperative AKI was 11.8% (range, 7.8%-15.9%). (Figure S10 in Supplementary Material). The in-hospital or 30-day mortality in patients with AKI was 2.44-fold higher than that in non-AKI patients (RR 3.12, 95%CI, 2.54–3.85, *p* < 0.01) (Figure 8).

### Publication bias

Inspection of the funnel plot for risk factor of prolonged CPB time were nearly symmetrical (Figure S11 in Supplementary Material), but the Egger's and Begg's *p* values were all <0.05, signifying significant publication bias (Table 2). But the bias was lost when trim and fill adjustment was performed (Figure S12 in Supplementary Material). Egger's and Begg's *p* values for other risk factors were also shown in Table 2. Begg’s *p* were all >0.05 even though Egger’s *p* < 0.05 for the risk factors of advanced age and increased pRBCs transfusion. In a word, the publication bias did not significantly affect the results of this meta-analysis.

**Table 2.** Begg’s and Egger’s *p* value of postoperative risks factors for acute kidney injury after Stanford type A aortic dissection repair surgery. Begg’s and Egger’s p value were all <0.05 for the risk factor of prolonged cardiopulmonary bypass time, and Begg’s p were >0.05 even though Egger's p < 0.05 for the risk factors of advanced age and increased packed red blood cells transfusion perioperatively.

Risk factors	Begg’s *p*-Value	Egger’s *p*-Value
Prolonged cardiopulmonary bypass time	0.05	<0.01
Prolonged operative time	0.12	0.32
Advanced age	0.12	0.04
Increased packed red blood cells transfusion perioperatively	0.17	0.004
elevated body mass index	0.12	0.13
preoperative kidney injury	0.14	0.05

## Discussion

Postoperative AKI is a common complication after TAAD repair surgery. This meta-analysis comprehensively analyzed that the synthesized incidence of postoperative AKI after TAAD repair surgery was 50.7%, which was consistent with previous range of 18–67% [3,4]. The previous range was relatively wide since different diagnostic criteria were used for AKI and heterogeneous TAAD patients underwent different repair surgeries, resulting in different incidence of postoperative AKI in different studies. However, in our meta-analysis, the most up-to-date and widely recognized diagnostic criteria with higher sensitivity of AKI diagnosis, that is KDIGO, was used in most studies, and the definition of postoperative AKI was same even though some studies used the diagnostic criteria of AKIN or RIFLE. Besides, in our meta-analysis, most patients with TAAD underwent total arch replacement repair surgery with deep hypothermia circulatory arrest (DHCA). Thus, most patients were relatively homogeneous and the slightly higher incidence of postoperative AKI of 50.7% was reasonable. The in-hospital mortality or 30-day mortality after surgery in patients with postoperative AKI was 11.8%, and patients with postoperative AKI had a 3.12-fold increased risk of mortality, which was consistent with the fact that AKI after TAAD repair surgery seriously affects the mortality of patients [5]. Under the circumstances, early identification of risk factors for AKI after TAAD repair surgery and preventing postoperative AKI are of great importance in improving outcomes.

Stanford type B aortic dissection may also involve renal arteries and lead to renal malperfusion, renal ischemia and injury, and the postoperative AKI may also be associated with age or weight of patients. However, the main surgical method for type B aortic dissection is thoracic endovascular aortic repair, which is obvious different from the open chest surgical repair with CPB for TAAD. Thus, the perioperative risk factors of postoperative AKI, such as operative time, CPB time, perioperative blood transfusions, are significantly different in type B aortic dissection repair from those in TAAD, so we did not include the patients of type B aortic dissection in this meta-analysis.

In fact, 30 items of risk factors for AKI after TAAD repair surgery were involved in data extraction. However, some risk factors were only concluded in one or two studies making it difficult to do further statistic. Hence only six risk factors were included in this meta-analysis, and all of them were statistically significant.

The present meta-analysis showed that prolonged CPB time, especially CPB time >180 min was a risk factor for AKI after TAAD repair, which was also consistent with previous meta-analysis [35] and most studies [5,6,14,16–20,23,25]. Roh *et al.* [6] found that the risk of AKI development after TAAD repair surgery is 4 times in patients with CPB time >180 min than in patients with CPB time <120 min. The CPB time >180 min not only increases 3 to 4 times of the risk of postoperative AKI, but also significantly increases the in-hospital mortality [36]. For Debakey type I aortic dissection, the incidence of AKI is increased by 17.1% for every 10 min added of CPB time, and after adjusting for the potential confounding factors by propensity score matching method, the results still remain statistically significant [19]. Meanwhile, it is obvious that the prolonged operative time is related to the prolonged CPB time. The underlying mechanism of the relationship between CPB time and AKI is unclear. The central pathogenesis of AKI during CPB are the reduced renal perfusion pressure, activation of pro-inflammatory mediators, direct nephrotoxicity and hemolysis [37], and all these factors may be aggravated as CPB and operative time extends. Longer CPB time and transfusions of autologous blood products from cell salvage devices may cause high levels of hemocytocatheresis and hemolysis during surgery [38]. Mamikonian *et al.* [39] also found that significant hemolysis occurs during cardiac surgery with CPB, which is related to the development of postoperative AKI. Therefore, reducing CPB-induced hemolysis and removing the free hemoglobin by endogenous mechanisms can minimize the toxic effect of acute hemolysis and therefore reduce the incidence of postoperative AKI. In addition, L. Lannemyr *et al.* [40] performed a research on the association between the renal tubular injury and CPB, and they found that the renal tubular cell injury is detected with a peak biomarker increased early after onset of CPB during cardiac surgery. The extent of renal tubular injury is independently associated with CPB time and rewarming quality. Therefore, shortening CPB time and avoiding hypothermia can decrease renal tubular cell injury. The CPB time is a modifiable factor. Qiu *et al.* [14] found that ascending aortic replacement combined with open triple-branched stent graft placement can reduce the occurrence of postoperative AKI and protect renal function *via* reducing CPB time. Thus, in patients with TAAD, reducing hemolysis, enhancing blood protection, simplifying surgical techniques, and shortening operation and CPB time, are considered to be important in protecting renal function and reducing the incidence of postoperative AKI.

Our study also showed that advanced age was a combined risk factor for AKI after TAAD repair surgery. This was consistent with the meta-analysis [7] and previous studies [14,17,23]. Helgason *et al.* and Zhou *et al.* found that for every 10 years of the increased age, the incidence of AKI after TAAD repair surgery is increased by 1.3 to 1.37 times [17,23], which may be owing to the poor renal function of elderly patients and poor renal ischemic tolerance to surgery with DHCA, leading to a higher incidence of postoperative AKI. The renal function gradually deteriorates with age [41]. In addition, elderly patients with AKI have a high rate of hospitalization, and are more likely to be affected by other comorbidities and are more likely to develop into chronic kidney disease [42]. However, Amano Kentaro *et al.* [43] suggested that younger patients are at a greater risk of postoperative AKI. The reason may be that some young patients with atherosclerosis or calcification of aortic dissection and the dissection can easily and widely tear from proximal aortic root to distal iliac artery or femoral artery, leading to a higher probability of total arch replacement repair, and resulting in an increased incidence of postoperative AKI. But we preferred to believe that advanced age is a risk factor for postoperative AKI according to our results. In a word, adequate attention should be paid to renal function of elderly patients after TAAD repair surgery.

The increased pRBCs transfusion perioperatively was also a combined risk factor for postoperative AKI. Qiu *et al.* [14] found that intraoperative and early postoperative RBC transfusions >10 units is a risk factor for early postoperative AKI after TAAD surgery. Kindzelski B. A *et al.* [44] found that intraoperative blood product transfusions are independently associated with an increased odds of developing AKI after cardiac surgery. Syed S. found that RBC transfusions above a threshold increases the incidence of postoperative complications and hospital length of stay among patients undergoing TAAD repair surgery [45]. The possible mechanism are RBC transfusions will cause pro-inflammatory reaction and increase oxidative stress, as well as result in the end-organ damage and adverse immunomodulatory effect on T-cell function [46]. Besides, hemolysis occurs during pRBCs storage and the following transfusions can lead to increased free hemoglobin and iron which will cause microcirculation dysfunction [47]. In addition, the need for pRBCs transfusion is also an indirect indicator of surgery complexity and more bleeding. Therefore, it is recommended to reduce operation-related bleeding and minimize pRBCs transfusion perioperatively in TAAD repair surgery.

Elevated BMI was a risk factor for AKI after TAAD repair surgery in our meta-analysis. The previous studies found that high BMI is an independent predictor of AKI in patients undergoing TAAD repair surgery [48] as well as other cardiovascular surgery with CPB [49]. High BMI may be associated with AKI development in critically ill patients [50] and it is also a risk factor for AKI in noncardiac surgery [51]. The association between elevated BMI and risk of AKI development could be multifactorial. First, obesity leads to glomerular hyperperfusion and hyperfiltration, and leads to deterioration of renal function called obesity-related glomerulopathy [52]. Second, obesity increases metabolic load on glomerulus, reducing functional nephrons [53]. Third, adipocytes can release inflammatory cytokines and cause oxidative stress in obese patients, and the increased oxidative stress can contribute to detrimental changes in glomerulus [54], which can be deteriorated by hypoperfusion, increased pro-inflammatory cytokines and microemboli during CPB. Fourth, excess abdominal fat increases abdominal pressure, which may cause renal dysfunction from both renal venous congestion and poor arterial perfusion. Fifth, as a component of metabolic syndrome, obesity is also a significant risk factor for cardiovascular disease, hypertension, and diabetes mellitus, which may be vulnerable to renal hypoperfusion during surgery with CPB. However, some study did not found that elevated BMI was a risk factor for postoperative AKI. Besides, Liu A *et al.* found that underweight Asian patients are susceptible to AKI in acute hospital settings [55]. The reason might be that malnutrition was independently associated with increased risks of AKI, morbidity, and mortality. In addition, nowadays, some studies found obesity paradox in some severe diseases. It refers to a phenomenon that being overweight or moderate obese was actually associated with superior clinical outcomes and a lower risk of all-cause mortality [56]. This may be associated with obese patients having higher nutrient, energy reserves and being able to cope with adverse reactions. However, obesity paradox has not been found in obesity undergoing TAAD repair surgery. Above all, we should pay close attention to postoperative renal function in obese patients undergoing TAAD surgery.

Preoperative kidney injury was also a risk factor of postoperative AKI. Preoperative malperfusion might be caused by cardiac tamponade and cardiogenic shock, or renal artery involvement owing to aortic dissection. Helgason et al. [23] found that the renal malperfusion is an independent risk factor for postoperative AKI, and 69% of patients with preoperative renal malperfusion developed AKI, usually be the most severe AKI stage. In the German Registry for Acute TAAD, Czerny and associates had shown that preoperative renal malperfusion increased the risk of postoperative renal ischemia by 11 times [57]. The reason between preoperative renal malperfusion and postoperative AKI is that the former leads to impaired preoperative renal function and poor renal ischemia tolerance. Besides, surgery with DHCA will lead to abnormal distribution of renal blood flow and increase renal vascular resistance [43], thus increasing the incidence of postoperative AKI. However, some study did not find that the preoperative renal malperfusion is a risk factor of postoperative AKI. The reason may be that renal perfusion was sustained by false lumen blood flow or was improved by true lumen blood flow as a result of the surgical repair. Another reason may be that renal malperfusion information is missing for 13% of patients, and some patients with renal hypoperfusion are neglected to be diagnosed as renal malperfusion due to different imaging quality [23]. So these reasons possibly resulted in an underestimation in the incidence of preoperative kidney injury. In conclusion, more attention should be paid to the patients with preoperative kidney injury.

In the present meta-analysis, DHCA was not associated with postoperative AKI. It may be related to the protective effects of DHCA on organ function, which counterbalance the kidney damage expected from prolonged CPB. Besides, compared with other cardiac surgeries, the TAAD repair surgery is more complex with longer CPB time and larger pRBCs transfusion, and the preoperative renal malperfusion is common. Thus these factors are more important than the factor of DHCA. But other investigations identified that DHCA is an independent risk factor for AKI [58]. These discrepancies may be explained to the confounding factors in heterogeneous cohorts.

## Conclusions

AKI was common after TAAD repair surgery, and it increased the in-hospital or 30-day mortality. The risk factors for postoperative AKI in the present meta-analysis included the prolonged CPB and operative time, advanced age, perioperative pRBCs transfusion, elevated BMI and preoperative kidney injury. The CPB time, operative time, and pRBCs transfusion was modifiable among the risk factors. Thus, improving surgical techniques and reducing CPB and operative time, and reducing pRBCs transfusion, can reduce the incidence of AKI after TAAD repair surgery and improve clinical outcomes, especially in elderly patients, patients with elevated BMI, or patients with preoperative kidney injury. Early identification of these risk factors enables clinicians to initiate efficient preventive and therapeutic strategies to reduce postoperative AKI.

## Strengths and limitations of this study

This meta-analysis performed a completed retrieval and a more focused analysis of risk factors for AKI after TAAD repair surgery, and the synthesized incidence of postoperative AKI and its impact on the in-hospital or 30-day mortality was analyzed. However, some risk factors are only concluded in one or two studies, so no further analysis was performed. As a result, some risk factors may be overlooked. Besides, long-term mortality, postoperative complications, threshold BMI value contributing to AKI were not analyzed. Moreover, the included articles were all retrospective studies, and this meta-analysis was not registered online, so randomized controlled trials are needed to identify risk factors so as to improve the clinical outcomes of patients undergoing TAAD repair surgery.

## Supplementary Material

Supplemental Material

## Data Availability

All data of this study are uploaded as online supplementary information.
