# Supplementary material for: Risk factors for acute kidney injury after Stanford type A aortic dissection repair surgery: a systematic review and meta-analysis
Source: Ren Fail. 2022 Aug 29;44(1):1462–76. doi: 10.1080/0886022X.2022.2113795 (PMC9427034; doi:10.1080/0886022X.2022.2113795)
Supplement: Supplemental Material [file IRNF_A_2113795_SM4708.pdf]

Supplementary materials

Supplementary Table 1 The medical subject heading terms of search strategy

		Pubmed	Embase	Web of science	Cochrane library
1#	(Aneurysm, Dissecting[Title/Abstract]) OR (Dissecting Aneurysm[Title/Abstract]) OR (Aneurysms, Dissecting[Title/Abstract]) OR (Dissecting Aneurysms[Title/Abstract]) OR (Dissection, Blood Vessel[Title/Abstract]) OR (Blood Vessel Dissection[Title/Abstract]) OR (Aortic Dissection[Title/Abstract]) OR (Aortic Dissections[Title/Abstract]) OR (Dissection, Aortic[Title/Abstract]) OR (Dissections, Aortic[Title/Abstract])	19680	24879	28859	1058
2#	(Acute Kidney Injury[Title/Abstract]) OR (Acute Kidney Injuries[Title/Abstract]) OR (Kidney Injuries, Acute[Title/Abstract]) OR (Kidney Injury, Acute[Title/Abstract]) OR (Acute Renal Injury[Title/Abstract]) OR (Acute Renal Injuries[Title/Abstract]) OR (Renal Injuries, Acute[Title/Abstract]) OR (Renal Injury, Acute[Title/Abstract]) OR (Renal Insufficiency, Acute[Title/Abstract]) OR (Acute Renal Insufficiencies[Title/Abstract]) OR (Renal Insufficiencies, Acute[Title/Abstract]) OR (Acute Renal Insufficiency[Title/Abstract]) OR (Kidney Insufficiency, Acute[Title/Abstract]) OR (Acute Kidney Insufficiencies[Title/Abstract]) OR (Kidney Insufficiencies, Acute[Title/Abstract]) OR (Acute Kidney Insufficiency[Title/Abstract]) OR (Kidney Failure, Acute[Title/Abstract]) OR (Acute Kidney Failures[Title/Abstract]) OR (Kidney Failures, Acute[Title/Abstract]) OR (Acute Renal Failure[Title/Abstract]) OR (Acute Renal Failures[Title/Abstract]) OR (Renal Failures, Acute[Title/Abstract]) OR (Renal Failure, Acute[Title/Abstract]) OR (Acute Kidney Failure[Title/Abstract])	73388	39285	96526	10327
3#	(Risk Factors[Title/Abstract]) OR (Factor, Risk[Title/Abstract]) OR (Factors, Risk[Title/Abstract]) OR (Risk Factor[Title/Abstract]) OR (Population at Risk[Title/Abstract]) OR (Risk, Population at[Title/Abstract]) OR (Populations at Risk[Title/Abstract]) OR (Risk, Populations at[Title/Abstract])	712780	1027126	1633711	130563
4#	1# AND 2# AND 3#	114	56	266	18

Supplementary Table 2 Inter-rater agreement at the screening stage.

Lei Wang	Xiaochai Lv	frequency
1	1	44
1	2	6
1	3	1
2	1	3
2	2	14
2	3	4
3	1	2
3	2	4
3	3	212

1：Studies met the inclusion criteria well.

2：Studies relatively met the inclusion criteria.

3：Studies did not meet the inclusion criteria.

Supplementary Table 3 Inter-rater agreement at the full-text review stage.

Lei Wang	Xiaochai Lv	frequency
1	1	19
1	2	1
1	3	0
2	1	2
2	2	2
2	3	2
3	1	0
3	2	1
3	3	30

1：Studies met the inclusion criteria well.

2：Studies relatively met the inclusion criteria.

3：Studies did not meet the inclusion criteria.

Supplementary Table 4 Quality assessment of the included studies [a]

	Selection				Comparability		Exposure			Total sores
Study	case definition	Representativeness of the cases	Controls Selection	Controls Definition	Controls for the most important factor	Controls for additional factor	Ascertainment of exposure	Same method of ascertainment for cases and controls	Non-Response rate	
Roh et al (2012)	*	*	*	*	*	*	*	*		8
Tsai et al (2012)	*	*	*	*		*		*	*	7
Hiraoka et al (2013)		*	*	*		*	*	*	*	7
Kim et al (2013)		*	*	*		*	*	*		6
Kim et al (2015)		*	*	*	*	*		*	*	7
Qiu et al (2015)	*	*	*	*		*	*			6
Ko et al (2015)	*	*	*	*	*	*		*	*	8
Ruan et al (2016)		*	*	*		*	*	*		6
Arnaoutakis et al (2016)	*	*	*	*	*	*	*	*	*	9
Zhou et al (2018)	*	*	*	*		*	*			6
Fang et al (2019)	*	*	*	*	*	*	*	*		8
Xu et al (2019)	*	*	*	*	*	*		*		7
Li L et al (2020)	*	*	*	*	*	*	*	*		8
Liu Y et al (2020)	*	*	*	*	*	*	*	*		8
Wang Z et al (2020)	*	*	*	*	*	*	*	*		8
Helgason et al (2021)	*		*	*	*	*	*		*	7
Tong et al (2021)	*	*	*	*	*		*	*		7
Ma et al (2021)	*	*			*		*	*		5
Li C et al (2022)	*	*	*	*	*	*	*	*		8
Yang et al (2022)	*	*		*	*		*	*		6

[a] Risk of bias was assessed using the Newcastle Ottawa Scale. A higher overall score indicated a lower risk of bias; a total score of ≥ 6 (of 9) was considered of high quality and low risk of bias.

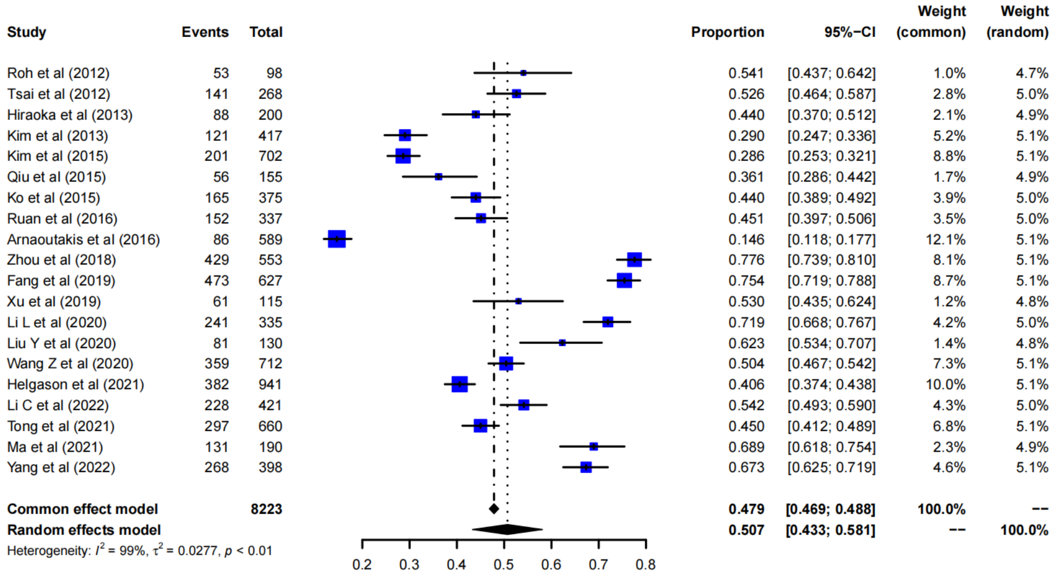

Supplementary figure 1 Forest plot of incidence of postoperative acute kidney injury after Stanford type A aortic dissection repair surgery. The solid squares are proportional to the weights used in the meta-analysis. The solid vertical line indicates no effect. The horizontal lines represent the 95% confifidence interval (CI). The diamond indicates the weighted odds ratio, and the lateral tips of the diamond indicate the associated 95% CI.

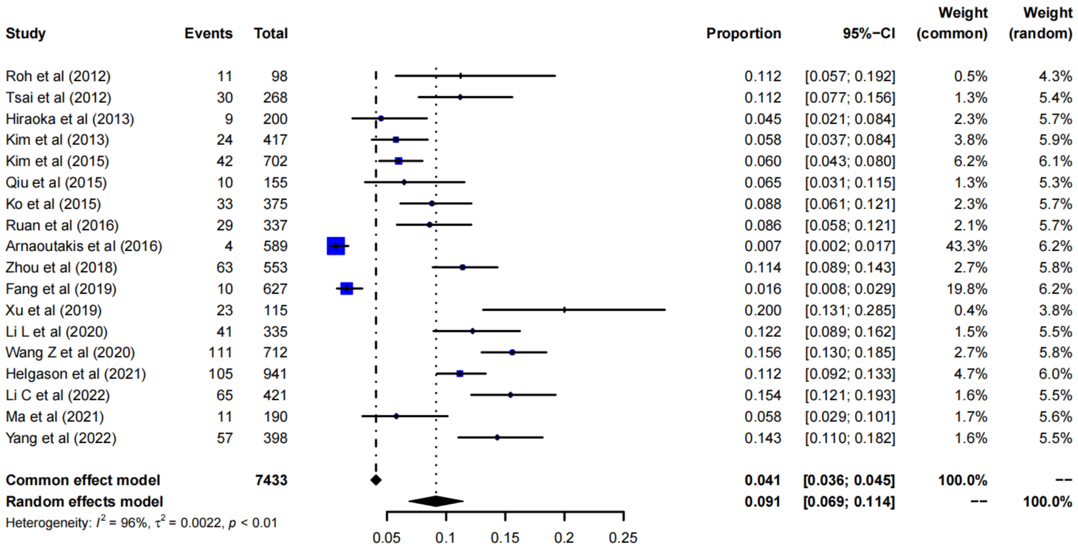

Supplementary figure 2 Forest plot of incidence of postoperative continuous renal replacement therapy after Stanford type A aortic dissection repair surgery. The solid squares are proportional to the weights used in the meta-analysis. The solid vertical line indicates no effect. The horizontal lines represent the 95% confifidence interval (CI). The diamond indicates the weighted odds ratio, and the lateral tips of the diamond indicate the associated 95% CI.

Study or Subgroup	Events	Total	Weight (common)	Weight (random)	IV, Fixed + Random, 95% CI
AKI Diagnosis = AKIN					
Qiu et al (2015)	56	155	1.7%	4.9%	0.36 [0.29; 0.44]
AKI Diagnosis = KDIGO					
Ko et al (2015)	165	375	3.9%	5.0%	0.44 [0.39; 0.49]
Ruan et al (2016)	152	337	3.5%	5.0%	0.45 [0.40; 0.51]
Zhou et al (2018)	429	553	8.1%	5.1%	0.78 [0.74; 0.81]
Fang et al (2019)	473	627	8.7%	5.1%	0.75 [0.72; 0.79]
Xu et al (2019)	61	115	1.2%	4.8%	0.53 [0.44; 0.62]
Li L et al (2020)	241	335	4.2%	5.0%	0.72 [0.67; 0.77]
Liu Y et al (2020)	81	130	1.4%	4.8%	0.62 [0.53; 0.71]
Wang Z et al (2020)	359	712	7.3%	5.1%	0.50 [0.47; 0.54]
Li C et al (2022)	228	421	4.3%	5.0%	0.54 [0.49; 0.59]
Tong et al (2021)	297	660	6.8%	5.1%	0.45 [0.41; 0.49]
Ma et al (2021)	131	190	2.3%	4.9%	0.69 [0.62; 0.75]
Yang et al (2022)	268	398	4.6%	5.1%	0.67 [0.62; 0.72]
Total (fixed effect, 95% CI)		4853	56.4%	--	0.61 [0.60; 0.62]
Total (random effects, 95% CI)			--	60.1%	0.60 [0.53; 0.67]
Heterogeneity: $Tau^2 = 0.0152$; $Chi^2 = 380.06$, df = 11 ($P < 0.01$); $I^2 = 97\%$					
AKI Diagnosis = RIFLE					
Roh et al (2012)	53	98	1.0%	4.7%	0.54 [0.44; 0.64]
Tsai et al (2012)	141	268	2.8%	5.0%	0.53 [0.46; 0.59]
Hiraoka et al (2013)	88	200	2.1%	4.9%	0.44 [0.37; 0.51]
Kim et al (2013)	121	417	5.2%	5.1%	0.29 [0.25; 0.34]
Kim et al (2015)	201	702	8.8%	5.1%	0.29 [0.25; 0.32]
Arnaoutakis et al (2016)	86	589	12.1%	5.1%	0.15 [0.12; 0.18]
Helgason et al (2021)	382	941	10.0%	5.1%	0.41 [0.37; 0.44]
Total (fixed effect, 95% CI)		3215	41.9%	--	0.30 [0.29; 0.32]
Total (random effects, 95% CI)			--	35.0%	0.37 [0.27; 0.48]
Heterogeneity: $Tau^2 = 0.0196$; $Chi^2 = 250.1$, df = 6 ($P < 0.01$); $I^2 = 98\%$					
**Total (fixed effect, 95% CI)**		**8223**	**100.0%**	**--**	**0.48 [0.47; 0.49]**
**Total (random effects, 95% CI)**			**--**	**100.0%**	**0.51 [0.43; 0.58]**

Heterogeneity: $Tau^2 = 0.0277$; $Chi^2 = 1525.73$, df = 19 ($P < 0.01$); $I^2 = 99\%$
Test for subgroup differences (fixed effect): $Chi^2 = 895.56$, df = 2 ($P < 0.01$)
Test for subgroup differences (random effects): $Chi^2 = 23.14$, df = 2 ($P < 0.01$)

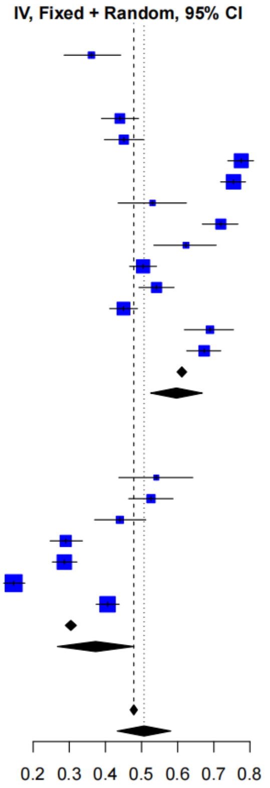

Supplementary figure 3 Forest plot of incidence of postoperative acute kidney injury after Stanford type A aortic dissection repair surgery grouped by diagnostic criteria of acute kidney injury. The solid squares are proportional to the weights used in the meta-analysis. The solid vertical line indicates no effect. The horizontal lines represent the 95% confifidence interval (CI). The diamond indicates the weighted odds ratio, and the lateral tips of the diamond indicate the associated 95% CI. Fixed, fixed effects; Random, random effects. RIFLE: Risk, Injury, Failure, Loss of function, End-stage renal disease; AKIN: Acute Kidney Injury Network; KDIGO: Kidney Disease Improving Global Outcomes; CPB: cardiopulmonary bypass;

Study or Subgroup	Events	Total	Weight (common)	Weight (random)	IV, Fixed + Random, 95% CI
**Country = China**					
Tsai et al (2012)	141	268	2.8%	5.0%	0.53 [0.46; 0.59]
Qiu et al (2015)	56	155	1.7%	4.9%	0.36 [0.29; 0.44]
Ruan et al (2016)	152	337	3.5%	5.0%	0.45 [0.40; 0.51]
Zhou et al (2018)	429	553	8.1%	5.1%	0.78 [0.74; 0.81]
Fang et al (2019)	473	627	8.7%	5.1%	0.75 [0.72; 0.79]
Xu et al (2019)	61	115	1.2%	4.8%	0.53 [0.44; 0.62]
Li L et al (2020)	241	335	4.2%	5.0%	0.72 [0.67; 0.77]
Liu Y et al (2020)	81	130	1.4%	4.8%	0.62 [0.53; 0.71]
Wang Z et al (2020)	359	712	7.3%	5.1%	0.50 [0.47; 0.54]
Li C et al (2022)	228	421	4.3%	5.0%	0.54 [0.49; 0.59]
Tong et al (2021)	297	660	6.8%	5.1%	0.45 [0.41; 0.49]
Ma et al (2021)	131	190	2.3%	4.9%	0.69 [0.62; 0.75]
Yang et al (2022)	268	398	4.6%	5.1%	0.67 [0.62; 0.72]
**Total (fixed effect, 95% CI)**		4901	57.0%	---	0.61 [0.60; 0.62]
**Total (random effects, 95% CI)**			---	64.9%	0.59 [0.51; 0.66]
Heterogeneity: $Tau^2 = 0.0164$; $Chi^2 = 385.18$, df = 12 ($P < 0.01$); $I^2 = 97\%$					
**Country = Japan**					
Hiraoka et al (2013)	88	200	2.1%	4.9%	0.44 [0.37; 0.51]
Ko et al (2015)	165	375	3.9%	5.0%	0.44 [0.39; 0.49]
**Total (fixed effect, 95% CI)**		575	6.0%	---	0.44 [0.40; 0.48]
**Total (random effects, 95% CI)**			---	10.0%	0.44 [0.40; 0.48]
Heterogeneity: $Tau^2 = 0$; $Chi^2 = 0$, df = 1 ($P = 1.00$); $I^2 = 0\%$					
**Country = Korea**					
Roh et al (2012)	53	98	1.0%	4.7%	0.54 [0.44; 0.64]
Kim et al (2013)	121	417	5.2%	5.1%	0.29 [0.25; 0.34]
Kim et al (2015)	201	702	8.8%	5.1%	0.29 [0.25; 0.32]
**Total (fixed effect, 95% CI)**		1217	15.0%	---	0.30 [0.28; 0.33]
**Total (random effects, 95% CI)**			---	14.9%	0.37 [0.21; 0.53]
Heterogeneity: $Tau^2 = 0.0187$; $Chi^2 = 23.59$, df = 2 ($P < 0.01$); $I^2 = 92\%$					
**Country = Pennsylvania or Iceland**					
Arnaoutakis et al (2016)	86	589	12.1%	5.1%	0.15 [0.12; 0.18]
Helgason et al (2021)	382	941	10.0%	5.1%	0.41 [0.37; 0.44]
**Total (fixed effect, 95% CI)**		1530	22.1%	---	0.26 [0.24; 0.28]
**Total (random effects, 95% CI)**			---	10.2%	0.28 [0.02; 0.53]
Heterogeneity: $Tau^2 = 0.0336$; $Chi^2 = 144.39$, df = 1 ($P < 0.01$); $I^2 = 99\%$					
**Total (fixed effect, 95% CI)**		8223	100.0%	--	0.48 [0.47; 0.49]
**Total (random effects, 95% CI)**			--	100.0%	0.51 [0.43; 0.58]
Heterogeneity: $Tau^2 = 0.0277$; $Chi^2 = 1525.73$, df = 19 ($P < 0.01$); $I^2 = 99\%$					
Test for subgroup differences (fixed effect): $Chi^2 = 972.57$, df = 3 ($P < 0.01$)					
Test for subgroup differences (random effects): $Chi^2 = 16.05$, df = 3 ($P < 0.01$)					

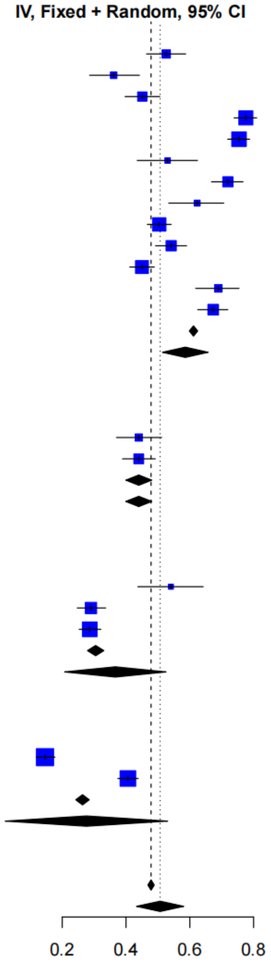

Supplementary figure 4 Forest plot of incidence of postoperative acute kidney injury after Stanford type A aortic dissection repair surgery grouped country. The solid squares are proportional to the weights used in the meta-analysis. The solid vertical line indicates no effect. The horizontal lines represent the 95% confifidence interval (CI). The diamond indicates the weighted odds ratio, and the lateral tips of the diamond indicate the associated 95% CI. Fixed, fixed effects; Random, random effects.

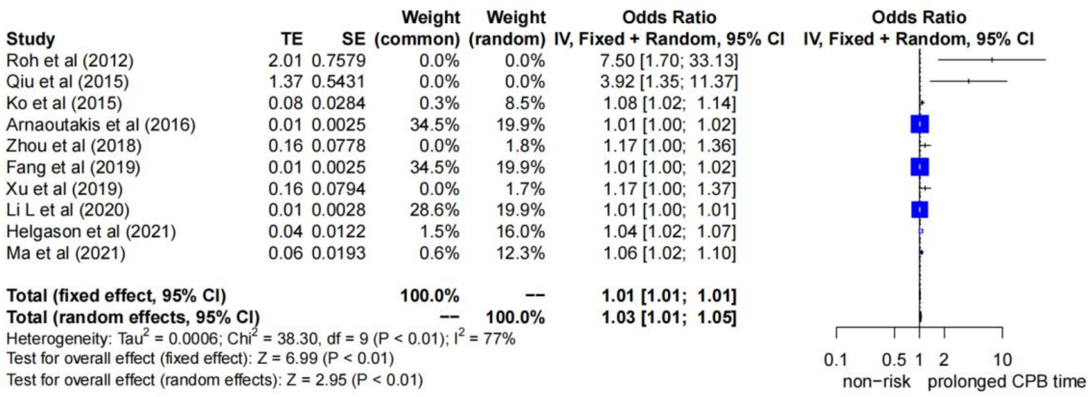

Supplementary figure 5 Forest plot of risk factor of prolonged CPB time. The solid squares are proportional to the weights used in the meta-analysis. The solid vertical line indicates no effect. The horizontal lines represent the 95% confifidence interval (CI). The diamond indicates the weighted odds ratio, and the lateral tips of the diamond indicate the associated 95% CI. CPB, cardiopulmonary bypass; Fixed, fixed effects; Random, random effects.

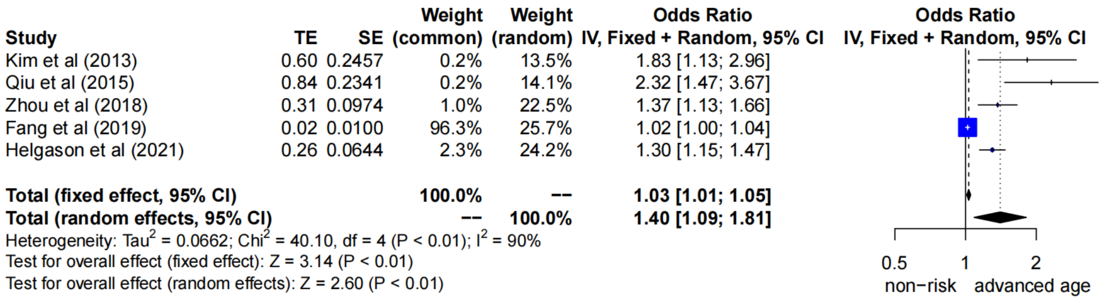

Supplementary figure 6 Forest plot of risk factor of advanced age. The solid squares are proportional to the weights used in the meta-analysis. The solid vertical line indicates no effect. The horizontal lines represent the 95% confifidence interval (CI). The diamond indicates the weighted odds ratio, and the lateral tips of the diamond indicate the associated 95% CI. Fixed, fixed effects; Random, random effects.

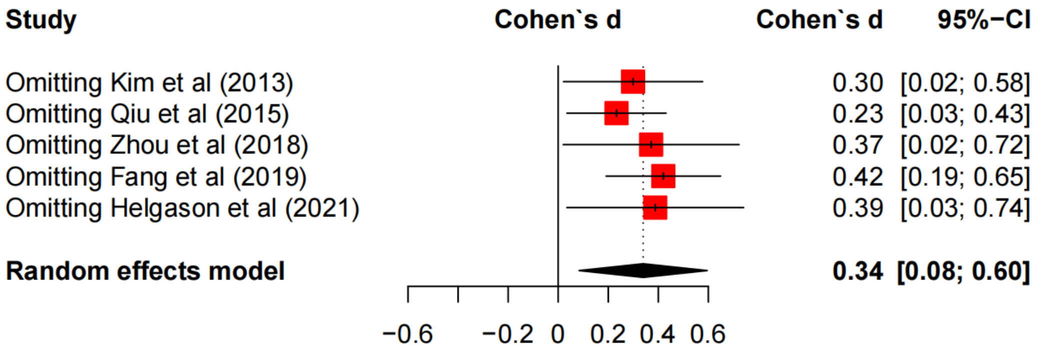

Supplementary figure 7 Sensitivity analysis of risk factors of advanced age.

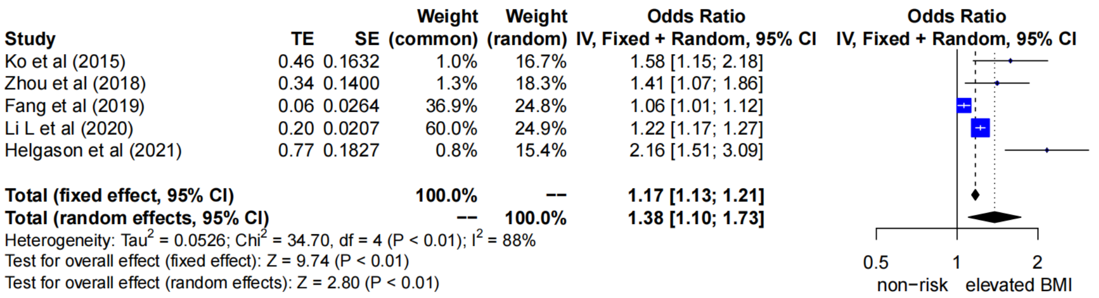

Supplementary figure 8 Forest plot of risk factor of elevated BMI. The solid squares are proportional to the weights used in the meta-analysis. The solid vertical line indicates no effect. The horizontal lines represent the 95% confifidence interval (CI). The diamond indicates the weighted odds ratio, and the lateral tips of the diamond indicate the associated 95% CI. BMI, body mass index, Fixed, fixed effects; Random, random effects.

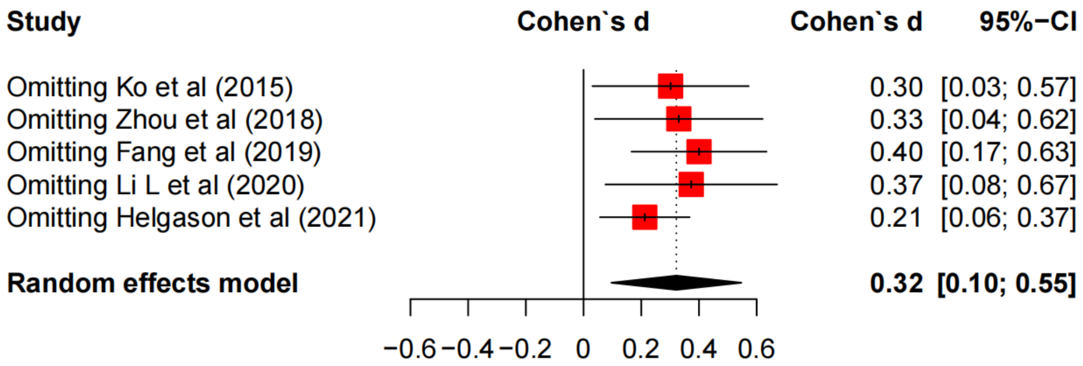

Supplementary figure 9 Sensitivity analysis of risk factor of elevated body mass index.

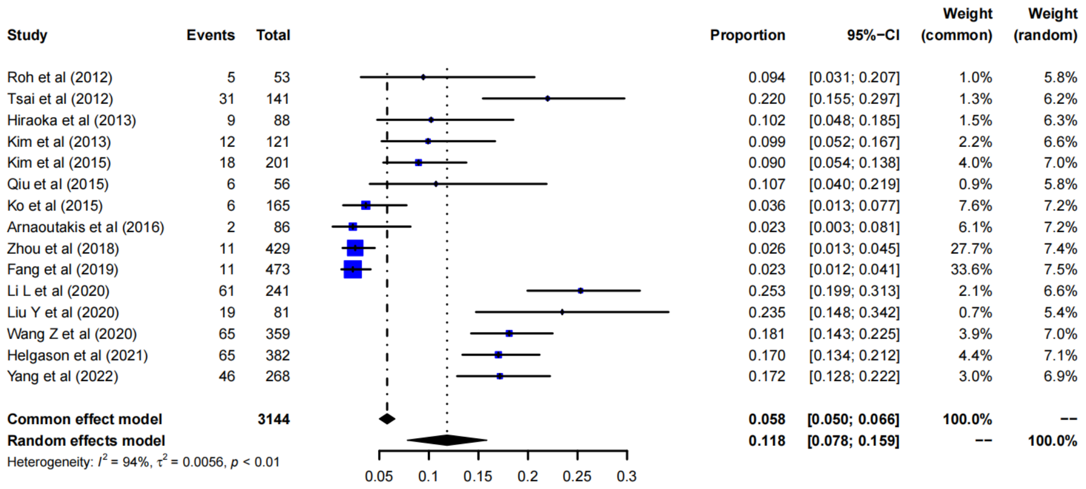

Supplementary figure 10 Forest plot of incidence of in-hospital and 30-day mortality in acute kidney injury group after TAAD repair surgery.

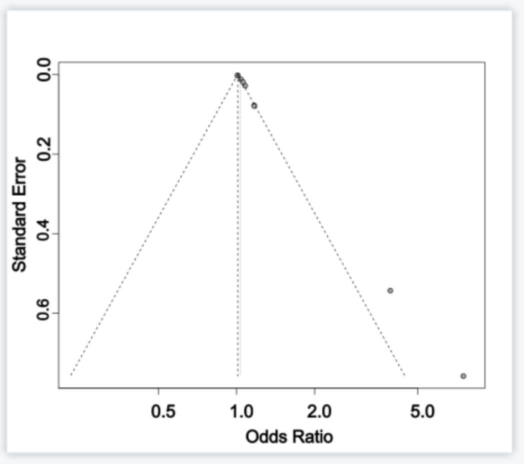

Supplementary figure 11 Funnel plot for risk factor of prolonged cardiopulmonary bypass time。

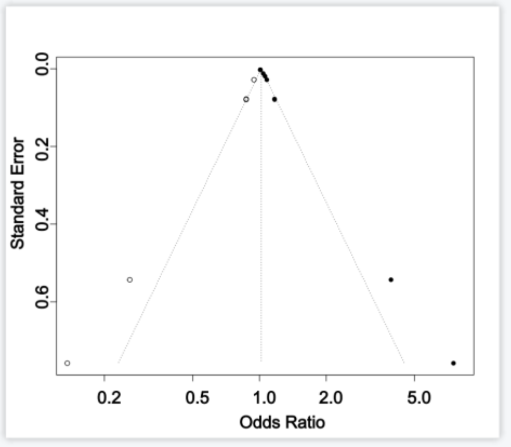

Supplementary figure 12 Trim and fill of risk factor for prolonged cardiopulmonary bypass time。
